# Epoxy Compositions with Reduced Flammability Based on DER-354 Resin and a Curing Agent Containing Aminophosphazenes Synthesized in Bulk Isophoronediamine

**DOI:** 10.3390/polym14173592

**Published:** 2022-08-31

**Authors:** Alexey Orlov, Anastasia Konstantinova, Roman Korotkov, Pavel Yudaev, Yaroslav Mezhuev, Ivan Terekhov, Leonid Gurevich, Evgeniy Chistyakov

**Affiliations:** 1 Department of Chemical Technology of Plastics, Mendeleev University of Chemical Technology of Russia, Miusskaya Sq. 9, 125047 Moscow, Russia; 2 Prepreg-ACM JSC 42, Bldg. 13, Volgogradskiy Prospekt, 109316 Moscow, Russia; 3 Department of Materials and Production, Aalborg University, Skjernvej 4A, 9220 Aalborg, Denmark

**Keywords:** epoxy resin, curing, phosphazene, noncombustibility, fire resistance, composition, mechanical properties

## Abstract

A method for the synthesis of an amine-containing epoxy resin curing agent by dissolving hexakis-[(4-formyl)phenoxy]cyclotriphosphazene in an excess of isophoronediamine was developed. The curing agent was characterized via NMR and IR spectroscopy and MALDI-TOF mass spectrometry, and its rheological characteristics were studied. Compositions based on DER-354 epoxy resin and the synthesized curing agent with different amounts of phosphazene content were obtained. The rheological characteristics of these compositions were studied, followed by their curing. An improvement in several thermal (DSC), mechanical (compression, tension, and adhesion), and physicochemical (water absorption and water solubility) characteristics, as well as the fire resistance of the obtained materials modified with phosphazene, was observed, compared with unmodified samples. In particular, there was an improvement in adhesive characteristics and fire resistance. Thus, compositions based on a curing agent containing a 30% modifier were shown to fulfill the V-1 fire resistance category. The developed compositions can be processed by contact molding, winding, and resin transfer molding (RTM), and the resulting material is suitable for use in aircraft, automotive products, design applications, and home repairs.

## 1. Introduction

Epoxy resins (ERs) are among the most important commercial thermosetting binders for the production of polymeric materials [1,2,3,4]. Due to having high-performance indicators such as processibility, physical and mechanical properties, chemical resistance, good adhesion to most known materials, and electrical insulating properties, epoxy binders and their derivatives are widely used in many industries and have various applications including in aviation, rocket and space industries, boatbuilding and shipbuilding, road and rail transport, sports equipment, medicine, the electronics industry, civil engineering, and green energy [5,6,7,8,9,10,11,12,13,14,15,16].

Despite the aforementioned advantages, epoxy binders and their derived products also have certain drawbacks, the main of which are high flammability and low fire resistance. These disadvantages limit the broader use of ERs and also increase the risk of their application in existing technologies. Therefore, one of the most important research tasks is to reduce combustibility in epoxy materials [17,18].

The fireproofing of epoxy materials is achieved by including special substance flame retardants into binders, which are conventionally categorized into additive and reactive types [19,20,21,22,23,24,25,26,27].

Additive flame retardants are widely used for improving the noncombustibility of both thermosetting and thermoplastic polymers. Their low cost, composition, and production simplicity have allowed them to gain a significant share of the flame retardant market. However, it should be noted that, in order to meet the modern fire safety requirements, a significant amount of flame retardant has to be added to the binder, which first and foremost significantly decreases the processibility and reduces the performance characteristics of the final product. The migration of an additive-type flame retardant during processing or curing is also not uncommon, which leads to the anisotropy of the product properties [28,29,30,31,32,33].

Unlike additive flame retardants, reactive flame retardants contain reactive groups within their structure, which allows them to be embedded into the three-dimensional network of a cured binder. For this reason, such flame retardants cannot migrate and exude from the binder or a manufactured product. Oftentimes, reactive flame retardants not only increase the fire resistance properties and reduce the flammability of epoxy resins [34,35,36,37,38,39,40,41,42,43,44] but also improve the physical and mechanical properties of the final product [45,46,47].

Both the modified epoxy resins [48,49,50,51,52,53,54,55,56,57,58,59,60,61] and curing agents [62,63,64,65,66] containing cyclotriphosphazene, phosphophenanthrene, phosphonate, and phosphate fragments can be used as reactive flame retardants for epoxy compositions. Compared with modified resins, curing agents containing flame retardants are easier to prepare and have a larger molecular design during synthesis. Recently, curing agents containing phosphorus-based flame retardants have become most widely used: P-modified Schiff bases, anhydrides, aliphatic amines, and imidazoles [67,68,69,70,71,72].

Among reactive flame retardants, phosphorus–nitrogen-containing compounds have a special place due to their environmentally friendly properties and a significant reduction in flammability due to their cooperative effects [73,74,75,76,77]. However, many of the presented compounds are crystalline solids that are insoluble in ERs. Combining such flame retardants with curing agents requires either high temperature or the use of a solution method. This significantly limits the application of epoxy materials with this type of flame retardant.

In this regard, the most rational technological approach is the use of curing agents containing a liquid phosphorus–nitrogen flame retardant [74,77]. At the same time, liquid phosphazene-containing curing agents are quite promising due to the simplicity of their chemical functionalization, which allows controlling the structure of phosphazene molecules and, accordingly, providing the necessary properties to both the phosphazenes themselves and the cured epoxy resins.

The present article describes the preparation of a liquid ER curing agent based on isophoronediamine (IPDA) and hexa-p-formylphenoxycyclotriphosphazene. The proposed method makes it possible to synthesize amine-containing aryloxyphosphazene in the IPDA bulk with different amounts of phosphazene content in the curing agent. The curing agent did not show a reduction in reactivity compared with the original IPDA and increased the fire resistance and performance of the cured DER-354 epoxy resin.

## 2. Materials and Methods

### 2.1. Materials

Hexachlorocyclotriphosphazene, 99% (Fushimi Pharmaceutical Co., Ltd., Maru-game, Kagawa Prefecture, Japan); 4-Hydroxybenzaldehyde, 98%; magnesium sulfate, anhydrous, ≥99.5%; chloroform, anhydrous, ≥99%; tetrahydrofuran, anhydrous, ≥99.9%; sodium, 99.9%; ethanol, anhydrous, ≥99.5%; epoxy resin DER-354, EEW = 170.63 gE, and isophoronediamine, AHEW = 43 gE (Sigma-Aldrich, Saint Louis, MO, USA).

### 2.2. Methods

^1^H and ^31^P NMR spectra were recorded on an Agilent/Varian Inova 400 spectrometer (Agilent Technologies, Santa Clara, CA, USA) at 400.02 MHz and 161.94 MHz, respectively.

IR spectra were recorded using a Nicolet 380 spectrometer (Thermo Fisher Scientific, Waltham, MA, USA) in the spectral range 4000–500 cm^−1^ with a wavenumber accuracy of 0.01 cm^−1^.

The glass transition temperature was determined according to ISO 11357-2:2020 using a Netzsch DSC 200F3 Maya differential scanning calorimeter (Erich NETZSCH GmbH and Co. Holding KG, Selb, Germany). The heating rate for all measurements was 10 K/min. All tests were performed in the temperature range of 30–300 °C in a nitrogen atmosphere at a flow rate of 40 mL/min.

The glass transition temperature and the development of the storage modulus, loss modulus, and tan δ mechanical losses were determined according to ASTM D7028-07(2015) using dynamic mechanical analysis on a Netzsch DMA 242E Artemis device (Erich NETZSCH GmbH and Co. Holding KG, Selb, Germany). Measurements were performed in a three-point bending mode within the temperature range of 30–200 °C at a standard heating rate of 5 K/min in a nitrogen atmosphere at a flow rate of 100 mL/min. The oscillation frequency was 1 Hz, and the load amplitude was 12 N during all the performed tests.

The gel time was determined according to the ISO 9396:1997 method. The automatic gel time determination for resins was carried out on a GELNORM GT-S gel timer (Gel Instrumente AG, Oberuzwil, Switzerland) with a plunger stroke rate of 10 s at room temperature.

The rheology properties of the obtained compounds were determined at 25 °C according to ISO 3219-1993 using a rotational viscometer on a Brookfield CAP 2000+ viscometer (AMETEK Brookfield, Middleboro, MA, USA) with a cone-plate geometry with CAP-1. All tests were performed with a constant shear rate depending on viscosity.

The tensile strength and tensile modulus were determined according to ISO 527-2:2012 on a 50ST Tinius Olsen universal testing machine (Tinius Olsen TMC, Horsham, PA, USA) with a traverse movement speed of 1 mm/min. The strains were measured using a video extensometer VEM208 (Tinius Olsen TMC, Horsham, PA, USA).

The compressive strength was estimated according to ISO 604:2002 on a 50ST Tinius Olsen universal testing machine (Tinius Olsen TMC, Horsham, PA, USA) with a punch movement speed of 1 mm/min.

The shear strength of the adhesive bond was determined according to ISO 4587-79 on a 50ST Tinius Olsen universal testing machine (Tinius Olsen TMC, Horsham, PA, USA) with a traverse speed of 10 mm/min. Steel plates were used as the bonded material. Steel grade St3, analogue: A57036 (USA), SS330 (Japan), DC03 (Germany).

The water absorption and water solubility content of the test compositions were determined according to ISO 62:2008 methods 2 and 3, respectively.

Resistance to combustion for the prepared compositions was determined according to the UL-94 test.

Statistical analysis: The average values of the performance characteristics of various samples were compared using two-way ANOVA followed by Tukey’s special analysis for multiple comparisons.

### 2.3. Synthesis of Hexakis-[(4-formyl)phenoxy]cyclotriphosphazene (FPP)

Hexakis-[4-formylphenoxy]cyclotriphosphazene was synthesized using the method described in a previous study [78].

4-Hydroxybenzaldehyde (7.32 g, 0.06 mol) was dissolved in ethanol (30 mL) in a three-necked flask equipped with a stirrer and a reflux condenser. After the complete dissolution of 4-hydroxybenzaldehyde, the alcohol solution of sodium ethylate, which was obtained via the dissolution of sodium (1.15 g, 0.05 mol) in ethanol (20 mL), was loaded in the flask. The reaction time was 10 min; then, ethanol was distilled off on a rotary evaporator in vacuum. The residue was dried in vacuum up to a constant weight. The yield of the product was quantitative.

4-Hydroxybenzaldehyde phenolate (8.64 g, 0.06 mol) was loaded in a three-necked flask equipped with a stirrer and a reflux condenser, and tetrahydrofuran (40 mL) was added. A solution of hexachlorocyclotriphosphazene (2.61 g, 0.0075 mol) in tetrahydrofuran (20 mL) was added to the dispersion formed during stirring. The time of reaction was 9 h during solvent boiling. When the process was complete, the reaction mixture was filtered off, and the mother liquor was evaporated on a rotary evaporator. The product was recrystallized from the ethanol–chloroform mixture. Yield: 4.52 g (70%).

### 2.4. Synthesis of the Modified Phosphazene-Containing Curing Agent Based on Isophoronediamine and FPP

FPP was dissolved in 40 g of IPDA at 100 °C in a 100 mL round-bottom flask equipped with a magnetic stirrer and a reflux condenser as per the formulations given in Table 1. Then, magnesium sulfate was added to the resulting mixture, and synthesis was carried out at the same temperature for 24 h. Lastly, the precipitate was separated in a centrifuge. The resulting liquid transparent mass was used without further purification.

### 2.5. Preparation of Epoxy Resin Compositions and Their Curing

DER-354 was pretreated under vacuum at 40 °C for 2 h. The resin sample was further cooled to room temperature and then dosed with 1.5 g of a curing agent in the amounts listed in Table 2, and the mixture was stirred until homogeneous. The finished compound was poured into molds for curing, stored for one day at 25 °C, and then for 4 h at 120 °C.

## 3. Results and Discussion

**Figure 1:**
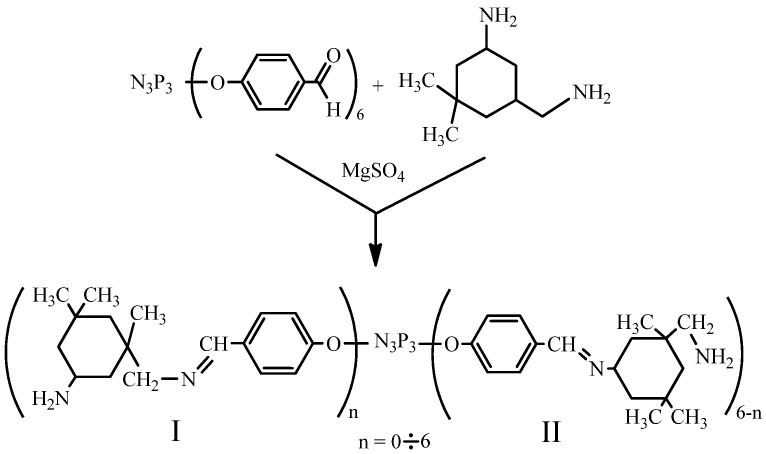
Scheme of reaction between FPP and IPDA.

The synthesis of the modified curing agent was carried out by the gradual dissolution of small portions of FPP in an excess of isophoronediamine to avoid the coalescence of the powder, which can significantly increase the dissolution time. The acceptor of the water released during the reaction was introduced into the solution only after the entire amount of FPP was completely dissolved since the heterogeneous particles of mineral salt prevented the dissolution of FPP. The general scheme of the reaction of FPP with IPDA is shown in Figure 1.

**Figure 2:**
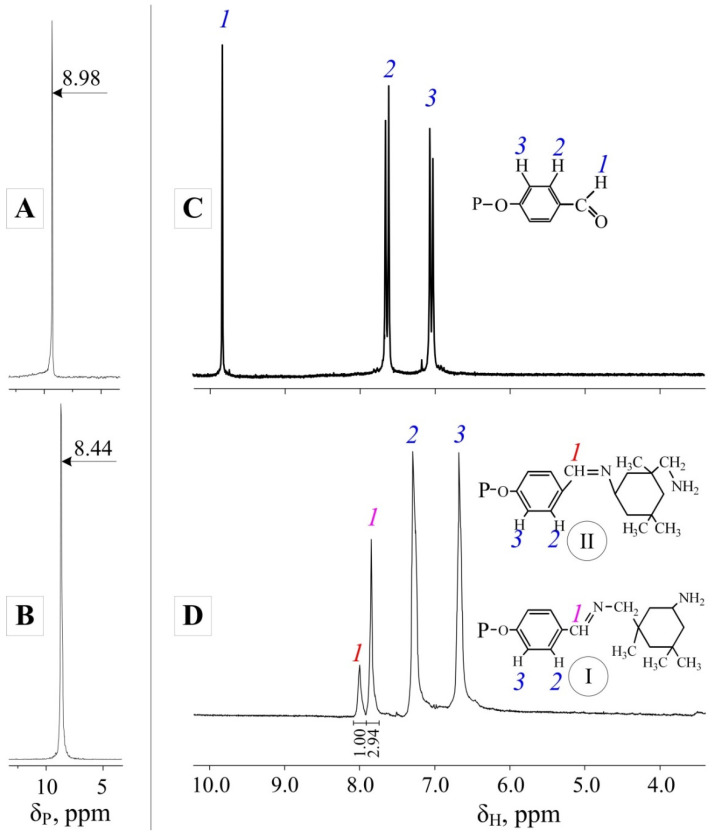
NMR spectra of ^31^P FPP (**A**) and its reaction product with IPDA (**B**), and ^1^H spectra of FPP (**C**) and its reaction product with IPDA (**D**).

It is not unreasonable to assume that since the IPDA molecule contains two different amino groups, the reaction will produce various azomethine derivatives of phosphazene. To confirm this, proton NMR spectroscopy of the modified curing agent was carried out. It can be seen from the spectrum (Figure 2D) that two types of azomethine fragments I and II were indeed formed (in accordance with the scheme presented in Figure 1). It should be noted that type I fragments were formed about three times more than type II fragments. Most likely, this is due to steric factors, since the amino group connected to the methylene fragment is further removed from the IPDA bulk cycle. In addition, comparing the spectra of the original FPP (Figure 2C) and those of the modified curing agent, it can be concluded that the aldehyde groups fully converted into azomethine groups.

Since the reaction between FPP and IPDA was carried out under rather aggressive conditions, it was necessary to investigate the product by means of phosphorus NMR spectroscopy. The spectrum of the modified curing agent showed a singlet at 8.44 ppm, indicating that the phosphazene ring remained unaffected (Figure 2B). However, there was a shift in the signal of the phosphorus atoms of the product by 0.54 ppm relative to the signal of the initial FPP (Figure 2A), which is due to the different degrees to which the mesomeric effects of aldehyde and azomethine groups influenced the phosphazene cycle. In this case, no splitting of the singlet was observed in the spectrum of the reaction product, which indicates that both fragments I and II had the same effect on phosphorus atoms.

**Figure 3:**
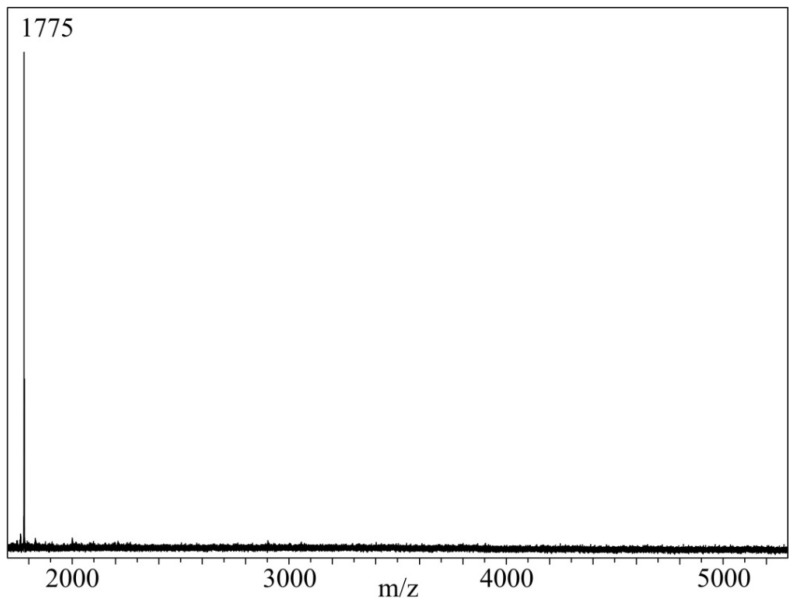
MALDI-TOF mass spectrum of the curing agent containing 30 wt. % FPP in IPDA.

Since FPP is a six-functional compound, and IPDA is a two-functional one, they can form branched, oligomeric, and polymeric products. To establish the presence of these products in the curing agent, MALDI-TOF mass spectrometry was performed. As can be seen from the spectrum (Figure 3) of the curing agent containing 30 wt.% of FPP, oligomeric compounds were not formed during the interaction of FPP and IPDA, since only a minor peak is present in the spectrum, corresponding to the mass of the condensation product of FPP with six IPDA molecules solvated by the proton of the matrix—1774 +H^+^. Most likely, this fact is due to a large molar excess of IPDA with respect to FPP, i.e., 17:1, which corresponds to about three IPDA molecules per one FPP carbonyl group. This, according to the Flory distribution, favors the formation of an individual compound. The steric factor caused by the bulkiness of the FPP molecules and the products of its condensation with IPDA should also not be excluded. In other samples, with a lower content of FPP and IPDA, the probability of the formation of oligomeric products is even lower.

Further, the characteristics of compositions based on the developed curing agent and DER-354 resin were studied. The choice of this brand of resin was due to the high viscosity of the modified curing agent and, consequently, the compositions derived from it, which makes it difficult to work with them. DER-354 is a medium-viscosity resin (3500 mPa∙s), and formulations based on it proved to be suitable for the study.

**Figure 4:**
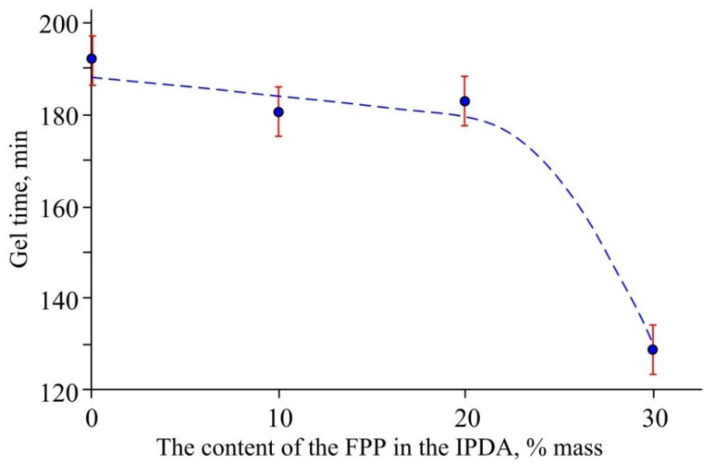
The gel time of the compositions vs. the FPP content in IPDA.

To assess the viability of the compositions, it was necessary to determine their gel time (Figure 4).

As can be seen from the plot, up to 20 wt.% of FPP in IPDA had practically no effect on the gel time of the compositions and averaged 3 h. However, at 30 wt.% FPP in IPDA, the gel time of the compositions was sharply reduced (by about an hour), which was visually related to the high viscosity of these compositions. Therefore, the dynamic viscosities of both the curing agents and the compositions based on them were investigated.

As per the results shown in Table 3, when the amounts of FPP in IPDA were 10, 20, and 30 wt.%, the viscosity of the curing agent increased by 5, 50, and 405 times, respectively, relative to pure IPDA. This can be explained by an increase in the number of bulk macromolecules of FPP azomethine derivatives in an IPDA solution, which have low mobility, as well as a simultaneous decrease in the content of IPDA itself.

As for the compositions, with an increase in the phosphazene content, their dynamic viscosity increased less than that in the case of curing agents. The viscosity of compositions with curing agents containing 10, 20, and 30 wt.% FPP increased by 2, 4, and 6 times, respectively, compared with the viscosity of a composition based on pure IPDA. At the same time, the viscosity of the compositions with curing agents containing up to 20 wt.% of FPP, inclusively, was lower than that of pure DER-354 (3500 mPa·s). In turn, the viscosity of the composition containing the curing agent with 30 wt.% FPP was 33% higher than that of pure resin. Obviously, the IPDA contained in the curing agents reduced the viscosity of the compositions, while the bulky molecules of azomethine derivatives of phosphazene caused an increase in viscosity. Based on the above, it can be concluded that a significant reduction in the gel time of the composition containing 30 wt.% FPP in the curing agent was due to the fact that the initial viscosity of the composition was higher with the introduction of the curing agent than that of the resin.

**Figure 5:**
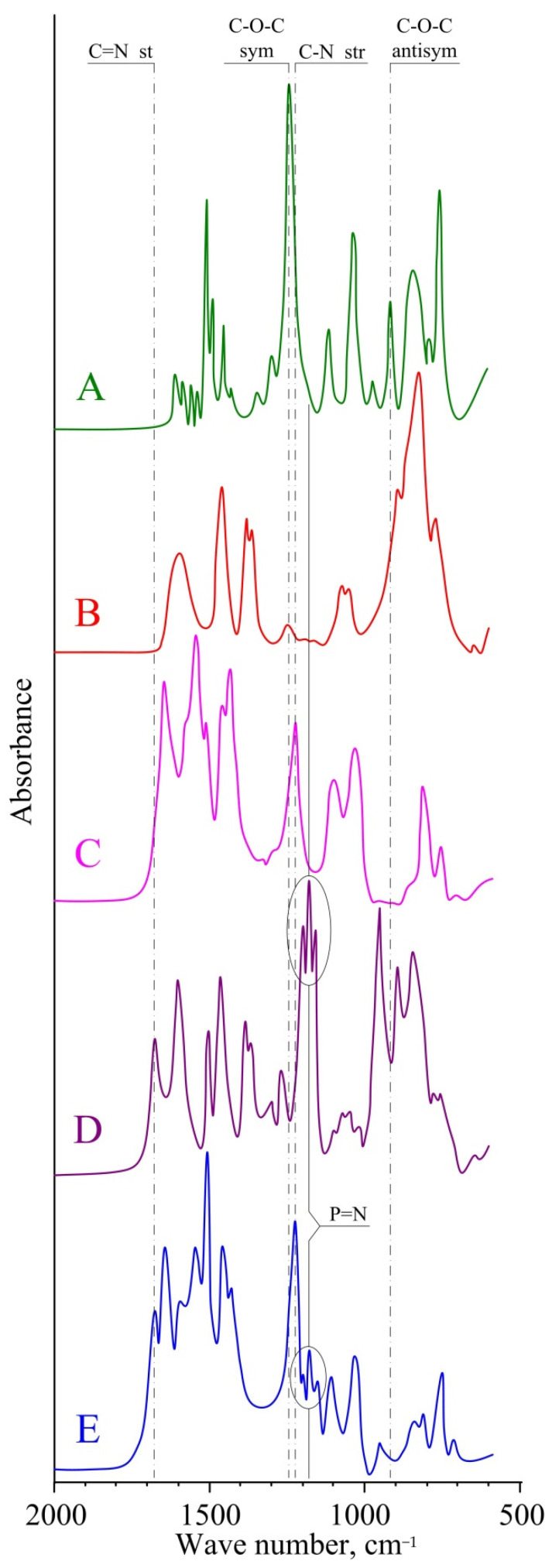
IR spectra: DER-354 (**A**); IPDA (**B**); DER-354 cured with pure IPDA (**C**); IPDA containing 30 wt.% FPP (**D**); DER-354 cured with IPDA containing 30 wt.% FPP (**E**).

The curing of epoxy compositions was carried out in two stages. Initially, the samples were kept for a day at room temperature to avoid the evaporation of IPDA. Then, the temperature was raised to 120 °C for the complete reaction between the epoxy and amino groups. To evaluate the conversion of epoxy groups during the curing of the resin, IR spectroscopy of the obtained samples was carried out. When comparing the IR spectra of DER-354 (Figure 5A) and those of the cured composition based on pure IPDA (Figure 5C), it can be seen that the band at 915 cm^−1^, present in the resin and characteristic of asymmetric vibrations of the oxirane ring, was not found in the composition. In addition, the band responsible for the symmetrical vibrations of the epoxy ring in the 1250 cm^−1^ region also disappeared. This indicates the complete conversion of the epoxy groups during the curing process. Instead, tertiary amino groups were formed, the corresponding signal for which appeared in the 1225 cm^−1^ region and was not observed earlier in the IPDA spectrum (Figure 5B). A similar pattern was observed in the spectrum of IPDA modified with phosphazene (Figure 5D) and in the spectrum of the composition cured with the modified curing agent (Figure 5E). In addition, it should be noted that, during the curing of the resin with a phosphazene-containing curing agent, the phosphazene cycle was retained, the vibration signals of which were in the region of 1220–1150 cm^−1^; the azomethine groups were also retained, and their stretch vibration signals were observed at 1680 cm^−1^.

The presence of the phosphazene modifier in the hardener affected the glass transition temperature of the studied compositions. As can be seen from Table 4, the Tg of the compositions cured with IPDA containing up to 20% modifier was slightly higher than that of compositions cured with pure IPDA. However, with 30% phosphazene in the hardener, the Tg of the cured composition dropped sharply. It can be assumed that this fact was due to the heterogeneity of the material structure due to steric factors associated with a high content of bulky azomethine derivatives of phosphazene (as in the case of viscosity).

A similar effect of the phosphazene content can be traced for most of the physical and mechanical characteristics of the cured compositions (Table 5). 

The modifier content had the greatest influence on the characteristics associated with the stretching of the cured compositions; specifically, it significantly increased their strength and modulus of elasticity. This can be explained by the fact that the substituents at the phosphorus atoms in phosphazene are linear polyconjugated structures, which determines their rigidity. Therefore, with a uniform distribution in the polymeric matrix, the phosphazene molecules, in the case of the stretching of the samples, act as reinforcing material [79,80]. In the case of the samples with the curing agent containing FPP 30 wt. %, the steric factor of the bulk molecules of the phosphazene curing agent and its high concentration contributed to the formation of inhomogeneous structures, which negatively affected the tensile strength.

The compression testing of the cured compositions showed that the modifier content had practically no effect on their characteristics. In this case, rigid phosphazene substituents did not have a significant reinforcing effect despite the fact that the phosphazene ring itself is quite flexible.

It is noteworthy that with an increase in the phosphazene content in the compositions, an increase in their adhesion to steel was also observed, which can be explained by an increase in the share of azomethine groups in the material composition, which are capable of coordinating many metals that make up the alloy. In all the cases, an adhesive gap was observed.

It should be noted that the modifier had practically no effect on the physicochemical properties of the compositions, such as water solubility and water absorption.

Fire resistance testing performed on horizontally fixed samples showed (Table 6) that all the samples containing the modifier did not form droplets of burning material.

At the same time, the burning rate of the samples decreased with an increase in the phosphazene content, and the composition based on the curing agent containing 30% FPP was self-extinguishing despite the fact that the mass content of phosphorus in them was below 1%. This indicator is quite high since greater fire resistance is achieved with a content of 4% phosphorus in the compositions. [81].

## 4. Conclusions

Despite the increased viscosity of the modified compositions relative to the unmodified composition, they may be suitable for processing into polymeric composite materials by methods such as contact molding, winding, and resin transfer molding (RTM).

Since the curing of epoxy resin with IPDA containing up to 20 wt.% of FPP contributed to an increase in the elastic modulus of the material by more than 40% and tensile strength by more than 20%, the area of low-load building blocks and housings is a promising application field for the developed compositions. Examples include internal and external transport elements such as small boat hulls, and automotive and railway products, which can be manufactured using the methods described above.

In turn, compositions based on the curing agent with 30% phosphazene, i.e., already at 0.86% phosphorus, are self-extinguishing and can be placed in the V-1 incombustibility category. There is a high demand for halogen-free polymeric materials with reduced flammability as interior binders for the transport industry, sheathing panels, floors, and seat elements. The developed compositions are also of interest as potting compounds for electrical and radio engineering devices. In addition, the compounds themselves can be used as dispersion-strengthened products obtained by casting, including pressure casting. These materials offer a high potential for reducing the probability of starting and spreading fires and can help to reduce life and property damage in the event of a fire.

The increased adhesion of the modified compositions to steel is promising for application in metal–polymer composites and repair of metal parts.

Thus, the developed compositions are promising binders for many areas of technology, such as road and rail transport, shipbuilding, aircraft building, household applications, and many more.

## Figures and Tables

**Table 1:** Curing agent composition formulations.

Ingredients	Weight of FPP Added to IPDA, wt. %		
	10	20	30
FPP, g	4	8	12
MgSO_4_, g	0.65	1.32	1.98

**Table 2:** The amount of resin dosed with the curing agent vs. the modifier content.

FPP Content in the Curing Agent, wt.%	DER-354 Weight, g
0	6.02
10	5.63
20	5.31
30	5.03

**Table 3:** Viscosity of the curing agent and the studied compositions vs. the modifier content.

Properties	FPP Content in IPDA, wt.%			
	0	10	20	30
Dynamic viscosity of the curing agent, mPa∙s	18	90	890	7300
Dynamic viscosity of the binder, mPa∙s	740	1320	2740	4650

**Table 4:** Dependence of the glass transition temperature of the cured compositions on the modifier content (the relative change with respect to the modifier-free samples is shown in brackets).

Parameter	FPP Content in IPDA, wt.%			
	0	10	20	30
Tg onset (DMA), °C	109	115 (+5.5%)	111 (+1.8%)	71 (−34.9%)
Tg end (DMA), °C	125	131 (+4.8%)	128 (+2.4%)	100 (−20.0%)
Tg (DSC), °C	119	121 (+1.7%)	121 (+1.7%)	89 (−25.2%)

**Table 5:** Dependence of the mechanical and physicochemical characteristics of the studied compositions on the modifier content in the hardener (relative change with respect relative to the modifier-free samples is shown in brackets).

Parameter	FPP Content in IPDA, wt.%			
	0	10	20	30
Tensile strength, MPa	40.7	52.7 (+29.5%)	50.7 (+24.6%)	38.8 (−4.7%)
Tensile modulus, MPa	1990	2820 (+41.7%)	2888 (+45.1%)	2570 (+29.1%)
Ultimate tensile strain, %	2.5	2.7 (+8.0%)	3.0 (+20%)	2.5 (0%)
Compressive strength, MPa	117.8	124.3 (+5.5%)	126.5 (+7.4%)	124.4 (+5.6%)
Compression modulus, MPa	1160	1120 (−3.4%)	1120 (−3.4%)	1120 (−3.4%)
Ultimate compression strain, %	14.0	13.7 (−2.1%)	13.7 (−2.1%)	14.9 (+6.4%)
Adhesion strength, MPa	4.43	4.61 (+4.1%)	4.67 (+5.4%)	4.71 (+9.5%)
Type of destruction	Adhesion	Adhesion	Adhesion	Adhesion
Water absorption, %	0.25	0.24 (−4.0%)	0.24 (−4.0%)	0.25 (0%)
Water solubility, %	0.12	0.12 (0%)	0.12 (0%)	0.12 (0%)

**Table 6:** Fire resistance test results for the cured DER-354.

FPP Content in IPDA, wt.%	Phosphorus Content in the Cured Resin, %	Burning Rate, mm/min.
0	0	17
10	0.26	15
20	0.51	13
30	0.86	10

## Data Availability

The data presented in this study are available on request from the corresponding author.

## References

[1] Cai R., Zhao J., Lv N., Fu A., Yin C., Song C., Chao M. (2022). Curing and Molecular Dynamics Simulation of MXene/Phenolic Epoxy Composites with Different Amine Curing Agent Systems. Nanomaterials.

[2] Rehman S., Gomez J., Villaro E., Cossey D., Karagiannidis P.G. (2022). Βio-Based Epoxy/Amine Reinforced with Reduced Graphene Oxide (rGO) or GLYMO-rGO: Study of Curing Kinetics, Mechanical Properties, Lamination and Bonding Performance. Nanomaterials.

[3] Merino E., Durán A., Ceré S., Castro Y. (2022). Hybrid Epoxy-Alkyl Sol–Gel Coatings Reinforced with SiO2 Nanoparticles for Corrosion Protection of Anodized AZ31B Mg Alloy. Gels.

[4] Decarpigny C., Ponchel A., Monflier E., Bleta R. (2021). Effect of Functional Group on the Catalytic Activity of Lipase B from Candida antarctica Immobilized in a Silica-Reinforced Pluronic F127/α-Cyclodextrin Hydrogel. Gels.

[5] Fang F., Ran S., Fang Z., Song P., Wang H. (2019). Improved Flame Resistance and Thermo-Mechanical Properties of Epoxy Resin Nanocomposites from Functionalized Graphene Oxide via Self-Assembly in Water. Compos. Part B-Eng..

[6] Min Y., Li P., Yin X., Ban D. (2017). Synthesis and Characterization of a Novel Flame Retardant Based on Phosphaphenanthrene for Epoxy Resin. Polym. Bull..

[7] Rad E.R., Vahabi H., de Anda A.R., Saeb M.R., Thomas S. (2019). Bio-Epoxy Resins with Inherent Flame Retardancy. Prog. Org. Coat..

[8] Vahabi H., Saeb M.R., Formela K., Lopez-Cuesta J.-M. (2018). Flame Retardant Epoxy/Halloysite Nanotubes Nanocomposite Coatings: Exploring Low-Concentration Threshold for Flammability Compared to Expandable Graphite as Superior Fire Retardant. Prog. Org. Coat..

[9] Xiong Y., Jiang Z., Xie Y., Zhang X., Xu W. (2013). Development of a DOPO-Containing Melamine Epoxy Hardeners and Its Thermal and Flame-Retardant Properties of Cured Products. J. Appl. Polym. Sci..

[10] Yan W., Yu J., Zhang M., Qin S., Wang T., Huang W., Long L. (2017). Flame-Retardant Effect of a Phenethyl-Bridged DOPO Derivative and Layered Double Hydroxides for Epoxy Resin. RSC Adv..

[11] Tian F., Cao J., Zhang S. (2022). Effect of Temperature on the Charge Transport Behavior of Epoxy/Nano−SiO_2_/Micro−BN Composite. Nanomaterials.

[12] Moghari S., Jafari S.H., Yazdi M.K., Jouyandeh M., Hejna A., Zarrintaj P., Saeb M.R. (2021). In-Out Surface Modification of Halloysite Nanotubes (HNTs) for Excellent Cure of Epoxy: Chemistry and Kinetics Modeling. Nanomaterials.

[13] Krieg A.S., King J.A., Odegard G.M., Leftwich T.R., Odegard L.K., Fraley P.D., Miskioglu I., Jolowsky C., Lundblad M., Park J.G. (2021). Mechanical properties and characterization of epoxy composites containing highly entangled as-received and acid treated carbon nanotubes. Nanomaterials.

[14] Tang Y., Tang C., Hu D., Gui Y. (2018). Effect of aminosilane coupling agents with different chain lengths on thermo-mechanical properties of cross-linked epoxy resin. Nanomaterials.

[15] Atta A.M., El-Newehy M.H., Abdulhameed M.M., Wahby M.H., Hashem A.I. (2021). Seawater absorption and adhesion properties of hydrophobic and superhydrophobic thermoset epoxy nanocomposite coatings. Nanomaterials.

[16] Xu Y.-J., Shi X.-H., Lu J.-H., Qi M., Guo D.-M., Chen L., Wang Y.-Z. (2020). Novel Phosphorus-Containing Imidazolium as Hardener for Epoxy Resin Aiming at Controllable Latent Curing Behavior and Flame Retardancy. Compos. Part B-Eng..

[17] Jin F.-L., Li X., Park S.-J. (2015). Synthesis and Application of Epoxy Resins: A Review. J. Ind. Eng. Chem..

[18] Zhu Z., Lin P., Wang H., Wang L., Yu B., Yang F. (2020). A Facile One-Step Synthesis of Highly Efficient Melamine Salt Reactive Flame Retardant for Epoxy Resin. J. Mater. Sci..

[19] Wu H., Zhang W., Zhang H., Gao P., Jin L., Pan Y., Pan Z. (2022). Synthesis of Layered Double Hydroxides with Phosphate Tailings and Its Effect on Flame Retardancy of Epoxy Resin. Polymers.

[20] Nageswara Rao T., Naidu T.M., Kim M.S., Parvatamma B., Prashanthi Y., Heun Koo B. (2019). Influence of zinc oxide nanoparticles and char forming agent polymer on flame retardancy of intumescent flame retardant coatings. Nanomaterials.

[21] Liu Q., Wang D., Li Z., Li Z., Peng X., Liu C., Zhang Y., Zheng P. (2020). Recent Developments in the Flame-Retardant System of Epoxy Resin. Materials.

[22] Chen R., Luo Z., Yu X., Tang H., Zhou Y., Zhou H. (2020). Synthesis of Chitosan-Based Flame Retardant and Its Fire Resistance in Epoxy Resin. Carbohydr. Polym..

[23] Zhu Z.-M., Wang L.-X., Lin X.-B., Dong L.-P. (2019). Synthesis of a Novel Phosphorus-Nitrogen Flame Retardant and Its Application in Epoxy Resin. Polym. Degrad. Stab..

[24] Cheng J., Wang J., Yang S., Zhang Q., Huo S., Zhang Q., Hu Y., Ding G. (2019). Benzimidazolyl-Substituted Cyclotriphosphazene Derivative as Latent Flame-Retardant Curing Agent for One-Component Epoxy Resin System with Excellent Comprehensive Performance. Compos. Part B-Eng..

[25] Kim M., Ko H., Park S.-M. (2019). Synergistic Effects of Amine-Modified Ammonium Polyphosphate on Curing Behaviors and Flame Retardation Properties of Epoxy Composites. Compos. Part B-Eng..

[26] Zhou X., Mu X., Cai W., Wang J., Chu F., Xu Z., Song L., Xing W., Hu Y. (2019). Design of Hierarchical NiCo-LDH@PZS Hollow Dodecahedron Architecture and Application in High-Performance Epoxy Resin with Excellent Fire Safety. ACS Appl. Mater. Interfaces.

[27] De la Cruz L.G., Abt T., León N., Wang L., Sánchez-Soto M. (2022). Ice-Template Crosslinked PVA Aerogels Modified with Tannic Acid and Sodium Alginate. Gels.

[28] Yao Z., Qian L., Qiu Y., Chen Y., Xu B., Li J. (2020). Flame Retardant and Toughening Behaviors of Bio-Based DOPO-Containing Curing Agent in Epoxy Thermoset. Polym. Adv. Technol..

[29] Wang H., Li S., Zhu Z., Yin X., Wang L., Weng Y., Wang X. (2021). A Novel DOPO-Based Flame Retardant Containing Benzimidazolone Structure with High Charring Ability towards Low Flammability and Smoke Epoxy Resins. Polym. Degrad. Stab..

[30] Duan H., Chen Y., Ji S., Hu R., Ma H. (2019). A Novel Phosphorus/Nitrogen-Containing Polycarboxylic Acid Endowing Epoxy Resin with Excellent Flame Retardance and Mechanical Properties. Chem. Eng. J..

[31] Sonnier R., Dumazert L., Livi S., Nguyen T.K.L., Duchet-Rumeau J., Vahabi H., Laheurte P. (2016). Flame Retardancy of Phosphorus-Containing Ionic Liquid Based Epoxy Networks. Polym. Degrad. Stab..

[32] Qiu Y., Qian L., Feng H., Jin S., Hao J. (2018). Toughening Effect and Flame-Retardant Behaviors of Phosphaphenanthrene/Phenylsiloxane Bigroup Macromolecules in Epoxy Thermoset. Macromolecules.

[33] Tan Y., Shao Z.-B., Chen X.-F., Long J.-W., Chen L., Wang Y.-Z. (2015). Novel Multifunctional Organic–Inorganic Hybrid Curing Agent with High Flame-Retardant Efficiency for Epoxy Resin. ACS Appl. Mater. Interfaces.

[34] Wang F., Liao J., Yan L., Cai M. (2022). Facile Construction of Polypyrrole Microencapsulated Melamine-Coated Ammonium Polyphosphate to Simultaneously Reduce Flammability and Smoke Release of Epoxy Resin. Polymers.

[35] Sun Y., Peng Y., Zhang Y. (2022). A Study on the Synthesis, Curing Behavior and Flame Retardance of a Novel Flame Retardant Curing Agent for Epoxy Resin. Polymers.

[36] Jiang J., Huo S., Zheng Y., Yang C., Yan H., Ran S., Fang Z. (2021). A Novel Synergistic Flame Retardant of Hexaphenoxycyclotriphosphazene for Epoxy Resin. Polymers.

[37] Wang F., Liao J., Yan L., Liu H. (2021). Fabrication of Diaminodiphenylmethane Modified Ammonium Polyphosphate to Remarkably Reduce the Fire Hazard of Epoxy Resins. Polymers.

[38] Qiu J.J., Xue Q., Liu Y.Y., Pan M., Liu C.M. (2014). A new polymer containing α-aminophosphonate unit used as reactive, halogen-free flame retardant for epoxy resins. Phosphorus Sulfur Silicon Relat. Elem..

[39] Szolnoki B., Toldy A., Marosi G. (2019). Effect of phosphorus flame retardants on the flammability of sugar-based bioepoxy resin. Phosphorus Sulfur Silicon Relat. Elem..

[40] Aljamal A., Marosi G., Szolnoki B. (2021). Flame retardancy effect of melamine cyanurate in combination with aluminum diethylphosphinate in a fully waterborne epoxy system. Phosphorus Sulfur Silicon Relat. Elem..

[41] Wang W., Li Y., Wei J., Luo Z., Pan C., Liu C. (2021). A novel polyhedral oligomeric silsesquioxanes derivative: Synthesis and characterization. J. Mol. Struct..

[42] Liang D., Zhu X., Dai P., Lu X., Guo H., Que H., Wang D., He T., Xu C., Robin H.M. (2021). Preparation of a novel lignin-based flame retardant for epoxy resin. Mater. Chem. Phys..

[43] Qian X., Song L., Bihe Y., Yu B., Shi Y., Hu Y., Yuen R.K. (2014). Organic/inorganic flame retardants containing phosphorus, nitrogen and silicon: Preparation and their performance on the flame retardancy of epoxy resins as a novel intumescent flame retardant system. Mater. Chem. Phys..

[44] Wang X., Song L., Xing W., Lu H., Hu Y. (2011). An effective flame retardant for epoxy resins based on poly (DOPO substituted dihydroxyl phenyl pentaerythritol diphosphonate). Mater. Chem. Phys..

[45] Wang P., Chen L., Xiao H., Zhan T. (2020). Nitrogen/Sulfur-Containing DOPO Based Oligomer for Highly Efficient Flame-Retardant Epoxy Resin. Polym. Degrad. Stab..

[46] Wang H., Zhu Z., Yuan J., Wang H., Wang Z., Yang F., Zhan J., Wang L. (2021). A New Recycling Strategy for Preparing Flame Retardants from Polyphenylene Sulfide Waste Textiles. Compos. Commun..

[47] Gu X., Huang X., Wei H., Tang X. (2011). Synthesis of Novel Epoxy-Group Modified Phosphazene-Containing Nanotube and Its Reinforcing Effect in Epoxy Resin. Eur. Polym. J..

[48] Sarychev I.A., Sirotin I.S., Borisov R.S., Mu J., Sokolskaya I.B., Bilichenko J.V., Filatov S.N., Kireev V.V. (2019). Synthesis of Resorcinol-Based Phosphazene-Containing Epoxy Oligomers. Polymers.

[49] Feng H., Wang X., Wu D. (2013). Fabrication of Spirocyclic Phosphazene Epoxy-Based Nanocomposites with Graphene via Exfoliation of Graphite Platelets and Thermal Curing for Enhancement of Mechanical and Conductive Properties. Ind. Eng. Chem. Res..

[50] Liu J., Tang J., Wang X., Wu D. (2012). Synthesis, Characterization and Curing Properties of a Novel Cyclolinear Phosphazene-Based Epoxy Resin for Halogen -Free Flame Retardancy and High Performance. RSC Adv..

[51] Peng W., Xu Y., Nie S., Yang W. (2021). A Bio-Based Phosphaphenanthrene-Containing Derivative Modified Epoxy Thermosets with Good Flame Retardancy, High Mechanical Properties and Transparency. RSC Adv..

[52] Wang H., Yuan J., Zhu Z., Yin X., Weng Y., Wang Z., Yang F., Zhan J., Wang H., Wang L. (2021). High Performance Epoxy Resin Composites Modified with Multifunctional Thiophene/Phosphaphenanthrene-Based Flame Retardant: Excellent Flame Retardance, Strong Mechanical Property and High Transparency. Compos. Part B-Eng..

[53] Liu Y.-L. (2002). Epoxy Resins from Novel Monomers with a Bis-(9,10-Dihydro-9-Oxa-10-Oxide-10-Phosphaphenanthrene-10-Yl-) Substituent. J. Polym. Sci. A Polym. Chem..

[54] Benin V., Cui X., Morgan A.B., Seiwert K. (2015). Synthesis and Flammability Testing of Epoxy Functionalized Phosphorous-Based Flame Retardants. J. Appl. Polym. Sci..

[55] Kim I.J., Ko J.W., Song M.S., Cheon J.W., Lee D.J., Park J.W., Yu S., Lee J.H. (2020). Thermal and Flame Retardant Properties of Phosphate-Functionalized Silica/Epoxy Nanocomposites. Materials.

[56] Wang X., Zhou S., Guo W.-W., Wang P.-L., Xing W., Song L., Hu Y. (2017). Renewable Cardanol-Based Phosphate as a Flame Retardant Toughening Agent for Epoxy Resins. ACS Sustain. Chem. Eng..

[57] Bornosuz N.V., Gorbunova I.Y., Kireev V.V., Bilichenko Y.V., Chursova L.V., Svistunov Y.S., Onuchin D.V., Shutov V.V., Petrakova V.V., Kolenchenko A.A. (2021). Synthesis and Application of Arylaminophosphazene as a Flame Retardant and Catalyst for the Polymerization of Benzoxazines. Polymers.

[58] Terekhov I.V., Chistyakov E.M., Filatov S.N., Kireev V.V., Buzin M.I. (2015). Hexa-Para-Aminophenoxycyclo-Triphosphazene as a Curing Agent/Modifier for Epoxy Resins. Int. Polym. Sci. Technol..

[59] Terekhov I.V., Chistyakov E.M., Filatov S.N., Deev I.S., Kurshev E.V., Lonskii S.L. (2019). Factors Influencing the Fire-Resistance of Epoxy Compositions Modified with Epoxy-Containing Phosphazenes. Inorg. Mater. Appl. Res..

[60] Sirotin I.S., Sarychev I.A., Filatov S.N., Kireev V.V., Terekhov I.V., Khaskov M.A. (2020). Physicomechanical Properties of Epoxy Composites Based on Low-Viscosity Phosphazene-Containing Epoxy-Resorcinol Resins. Polym. Sci. Ser. B.

[61] Salmeia K.A., Gaan S. (2015). An Overview of Some Recent Advances in DOPO-Derivatives: Chemistry and Flame Retardant Applications. Polym. Degrad. Stab..

[62] Jian R.-K., Ai Y.-F., Xia L., Zhao L.-J., Zhao H.-B. (2019). Single Component Phosphamide-Based Intumescent Flame Retardant with Potential Reactivity towards Low Flammability and Smoke Epoxy Resins. J. Hazard. Mater..

[63] Zhang Q., Yang S., Wang J., Cheng J., Zhang Q., Ding G., Hu Y., Huo S. (2019). A DOPO Based Reactive Flame Retardant Constructed by Multiple Heteroaromatic Groups and Its Application on Epoxy Resin: Curing Behavior, Thermal Degradation and Flame Retardancy. Polym. Degrad. Stab..

[64] Yang J.W., Wang Z.Z. (2017). Thermal and flame retardant properties of epoxy resin cured by a novel phosphorus-containing 4, 4′-bisphenol novolac curing agent. Phosphorus Sulfur Silicon Relat. Elem..

[65] Xiao L., Sun D.C., Niu T.L., Yao Y.W. (2014). Syntheses of two dopo-based reactive additives as flame retardants and co-curing agents for epoxy resins. Phosphorus Sulfur Silicon Relat. Elem..

[66] Sun Z., Hou Y., Hu Y., Hu W. (2018). Effect of additive phosphorus-nitrogen containing flame retardant on char formation and flame retardancy of epoxy resin. Mater. Chem. Phys..

[67] Liang B., Hong X.D., Wang C.S. (2012). Synthesis and Properties of a Novel Phosphorous-containing Flame-retardant Hardener for Epoxy Resin. J. Appl. Polym. Sci..

[68] Wirasaputra A., Yao X., Zhu Y., Liu S., Yuan Y., Zhao J., Fu Y. (2016). Flame-Retarded Epoxy Resins with a Curing Agent of DOPO-Triazine Based Anhydride. Macromol. Mater. Eng..

[69] Shao Z.-B., Zhang M.-X., Li Y., Han Y., Ren L., Deng C. (2018). A Novel Multi-Functional Polymeric Curing Agent: Synthesis, Characterization, and Its Epoxy Resin with Simultaneous Excellent Flame Retardance and Transparency. Chem. Eng. J..

[70] Xu Y.-J., Chen L., Rao W.-H., Qi M., Guo D.-M., Liao W., Wang Y.-Z. (2018). Latent Curing Epoxy System with Excellent Thermal Stability, F.; lame Retardance and Dielectric Property. Chem. Eng. J..

[71] Xie W., Huang S., Tang D., Liu S., Zhao J. (2020). Synthesis of a Furfural-Based DOPO-Containing Co-Curing Agent for Fire-Safe Epoxy Resins. RSC Adv..

[72] Buravov B.A., Bochkarev E.S., Al-Khamzawi A., Tuzhikov O.O., Tuzhikov O.I. (2020). Modern trends in the development of antipyrine for polymer compositions. Composition, properties, application. IZVESTIA VGTU.

[73] Li A., Mao P., Liang B. (2019). The Application of a Phosphorus Nitrogen Flame Retardant Curing Agent in Epoxy Resin. e-Polymers.

[74] Ma H., Tong L., Xu Z., Fang Z., Jin Y., Lu F. (2007). A Novel Intumescent Flame Retardant: Synthesis and Application in ABS Copolymer. Polym. Degrad. Stab..

[75] Wang Q., Chen Y., Liu Y., Yin H., Aelmans N., Kierkels R. (2004). Performance of an Intumescent-Flame-Retardant Master Batch Synthesized by Twin-Screw Reactive Extrusion: Effect of the Polypropylene Carrier Resin. Polym. Int..

[76] Yang X., Wang C., Xia J., Mao W., Li S. (2017). Study on Synthesis of Novel Phosphorus-Containing Flame Retardant Epoxy Curing Agents from Renewable Resources and the Comprehensive Properties of Their Combined Cured Products. Prog. Org. Coat..

[77] Huo S., Yang S., Wang J., Cheng J., Zhang Q., Hu Y., Ding G., Zhang Q., Song P. (2020). A Liquid Phosphorus-Containing Imidazole Derivative as Flame-Retardant Curing Agent for Epoxy Resin with Enhanced Thermal Latency, Mechanical, and Flame-Retardant Performances. J. Hazard. Mater..

[78] Chistyakov E.M., Kireev V.V., Filatov S.N., Terekhov I.V., Buzin M.I., Komarova L.I. (2012). Thermal polycondensation of hexa-p-hydroxymethylphenoxycyclotriphosphazene. Polym. Sci. Ser. B.

[79] Zhou X., Qiu S., He L., Wang X., Zhu Y., Chu F., Wang B., Song L., Hu Y. (2021). Synthesis of star-shaped allyl phosphazene small molecules for enhancing fire safety and toughness of high performance BMI resin. Chem. Eng. J..

[80] Abu-Shanab O.L., Chang C.P., Soucek M.D. (1996). Polyphosphazene toughened PMR-type thermosets. High Perform. Polym..

[81] Howell B.A., Lienhart G.W., Livingstone V.J., Aulakh D. (2020). 1-Dopyl-1, 2-(4-hydroxyphenyl) ethene: A flame retardant hardner for epoxy resin. Polym. Degrad. Stab..

